# TLR2 variants and *Helicobacter pylori*: revisiting a controversial link

**DOI:** 10.48101/ujms.v130.13533

**Published:** 2025-12-22

**Authors:** Duygu Kirkik, Sevgi Demircioglu, Sevgi Kalkanli Taş

**Affiliations:** a Department of Immunology, Hamidiye Faculty of Medicine, University of Health Sciences, Istanbul, Turkiye; b Department of Medical Biology, Hamidiye Faculty of Medicine, University of Health Sciences, Istanbul, Turkiye; c Department of Computer Engineering, Faculty of Engineering, Istanbul Arel University, Istanbul, Turkiye

**Keywords:** TLR2, *Helicobacter pylori*, del –196 to –174, meta-analysis, random-effects model

## Abstract

**Objective:**

Although polymorphisms in the Toll-like receptor 2 (TLR2) gene have been proposed as host genetic factors influencing susceptibility to *Helicobacter pylori* infection, existing data remain inconclusive. This meta-analysis aimed to clarify whether two common variants – rs3804099 and del –196 to –174 – contribute to infection risk across diverse populations.

**Materials and methods:**

A systematic search of PubMed, Scopus, and Web of Science (up to January 2025) identified eligible case–control studies examining the association between TLR2 polymorphisms and *H. pylori* infection. Pooled odds ratios (ORs) with 95% confidence intervals (CIs) were calculated using random-effects models. Heterogeneity, publication bias, and sensitivity were assessed according to PRISMA 2020 guidelines.

**Results:**

Ten studies comprising 4,521 subjects were included. Pooled analyses under allelic, dominant, recessive, homozygous, and heterozygous models revealed no significant association between either rs3804099 or del –196 to –174 polymorphisms and infection risk. Substantial inter-study heterogeneity was observed, particularly for rs3804099, but sensitivity analyses confirmed the stability of pooled results.

**Conclusion:**

This meta-analysis refutes a consistent genetic association between TLR2 rs3804099 or del –196 to –174 polymorphisms and *H. pylori* infection. The findings suggest that host innate immunity variability alone does not explain differences in infection susceptibility among populations. Future studies integrating bacterial virulence genotypes and host immunogenetic profiles are warranted to delineate population-specific risk mechanisms.

## Introduction

Helicobacter pylori (*H. pylori*) is a Gram-negative, spiral-shaped, microaerophilic bacterium that colonizes the human gastric epithelium, establishing chronic infection in more than 50% of the global population (1). Although many infected individuals remain asymptomatic, *H. pylori* are etiologically linked to several gastrointestinal pathologies, including chronic gastritis, peptic ulcer disease, gastric mucosa-associated lymphoid tissue (MALT) lymphoma, and non-cardia gastric adenocarcinoma (2–5). Epidemiological evidence suggests that *H. pylori* infection contributes to the pathogenesis of gastric cancer through a multistep process involving chronic inflammation, gastric atrophy, intestinal metaplasia, and dysplasia (6, 7).

The clinical heterogeneity observed among *H. pylori*-infected individuals can be attributed to differences in bacterial virulence factors (such as CagA and VacA), environmental exposures (diet, smoking, hygiene), and host genetic factors (8–10). One of the most critical aspects of host defense against *H. pylori* is the innate immune system, particularly through pattern recognition receptors such as Toll-like receptors (TLRs) (11). TLRs recognize pathogen-associated molecular patterns (PAMPs) and initiate signaling cascades that result in the activation of nuclear factor-kappa B (NF-κB), triggering the production of pro-inflammatory cytokines such as interleukin-8 (IL-8), tumor necrosis factor-alpha (TNF-α), and macrophage inflammatory proteins (12). Among these receptors, TLR2 has been shown to play a prominent role in the immune response to *H. pylori.* It recognizes bacterial lipoproteins, lipoteichoic acid, and peptidoglycans, facilitating immune activation in gastric epithelial and immune cells (13). *H. pylori* infection upregulates TLR2 expression and activates the NF-κB pathway through TLR2 engagement, which is thought to influence both bacterial clearance and disease severity (13, 14). Polymorphisms in the TLR2 gene may affect its expression or function, thereby modulating host responses to infection. Two well-studied variants in this context are the rs3804099 single nucleotide polymorphism (SNP) and the –196 to –174 deletion polymorphism (del) (15, 16). The rs3804099 variant is a synonymous SNP located in exon 3 of the TLR2 gene. Although it does not alter the amino acid sequence, studies suggest it may influence mRNA stability, splicing, or translation efficiency, ultimately impacting receptor expression (17). The –196 to –174del polymorphism, located in the promoter region, has been shown to reduce promoter activity and subsequently TLR2 expression levels, potentially impairing innate immune recognition of *H. Pylori* (18)*.* Several case–control studies have investigated the association between these polymorphisms and susceptibility to *H. pylori* infection, with conflicting results (19–21). For instance, Mirkamandar et al. found that the CT genotype of rs3804099 was significantly more prevalent in Iranian individuals infected with *H. pylori*, particularly those harboring cagA-positive strains, compared with uninfected controls (15). This suggested a possible genetic predisposition mediated by TLR2 variation. In contrast, a large-scale Japanese study by Hishida et al. reported no significant association between the –196 to –174del polymorphism and *H. pylori* seropositivity, gastric atrophy, or gastric cancer, emphasizing the potential influence of ethnic and regional genetic backgrounds (22). This study aims to elucidate the potential contribution of TLR2-mediated innate immunity to *H. pylori*-related disease outcomes and to explore possible sources of heterogeneity such as ethnicity, genotyping methods, and infection status (e.g. cagA-positive vs. negative strains). Through this investigation, we hope to clarify the role of TLR2 genetic variability in the host–pathogen interaction and its relevance to personalized disease prevention strategies.

## Materials and methods

This meta-analysis was conducted in accordance with the PRISMA 2020 (Preferred Reporting Items for Systematic Reviews and Meta-Analyses) guidelines (Supplementary File S1) (23–25). A comprehensive literature search was performed in PubMed, Scopus, and Web of Science databases to identify eligible studies published up to January 2025. The search strategy included a combination of keywords and MeSH terms such as ‘Toll-like receptors’, ‘TLR2’, ‘single nucleotide polymorphism’, ‘polymorphism’, ‘variant’, ‘genotype’, and ‘Helicobacter pylori infection’. No language or publication date restrictions were applied. The search results were screened and evaluated according to pre-defined inclusion and exclusion criteria.

### Eligibility criteria

Studies were deemed eligible for inclusion in this meta-analysis if they met the following criteria: 1) employed a case–control design investigating the relationship between the TLR2 rs3804099 or TLR2 del –196 to –174 polymorphisms and susceptibility to *Helicobacter pylori* infection; 2) provided sufficient genotype frequency data to allow for the calculation of effect sizes, either reported directly in the manuscript or derivable from available information; and 3) reported Hardy–Weinberg equilibrium (HWE) status for the genotype distributions in the control group, ensuring genetic validity. Studies were excluded if they did not conform to a case–control design, presented duplicate findings or insufficient data, or if multiple publications from the same research group used overlapping datasets – in which case, only the study with the largest sample size and most comprehensive data was retained for analysis. These criteria were applied rigorously to ensure the inclusion of high-quality, non-redundant, and methodologically appropriate studies.

### Data extraction and quality assessment

Data extraction was independently conducted by two investigators in accordance with the eligibility criteria. Discrepancies between reviewers were resolved through discussion and consensus. Extracted data included: the first author’s name, year of publication, country or ethnicity of the study population, sample sizes for case and control groups, HWE status in controls, average age, sex distribution, and genotype frequencies of the relevant TLR2 polymorphisms in both groups.

### Statistical analysis

Meta-analytical computations were performed using R software (version 4.4.2), employing the ‘meta’, metafor’, and ‘dmetar’ packages (26, 27). The associations between the TLR2 rs3804099 and TLR2 del –196 to –174 polymorphisms and *H. pylori* infection risk were assessed under five genetic models: allelic, dominant, recessive, homozygous, and heterozygous. Pooled odds ratios (ORs) and 95% confidence intervals (CIs) were calculated for each model.

Statistical heterogeneity among studies was assessed using the Cochran Q test and quantified with the I² statistic. A *P* < 0.05 or *I*² > 50% was considered indicative of significant heterogeneity. In such cases, a random-effects model (Der Simonian and Laird method) was applied (28). Potential publication bias was examined using Egger’s linear regression test, and funnel plots were visually inspected. To evaluate the robustness of the findings, sensitivity analyses were conducted by sequentially excluding individual studies.

## Results

### Study selection and characteristics

**Figure 1:**
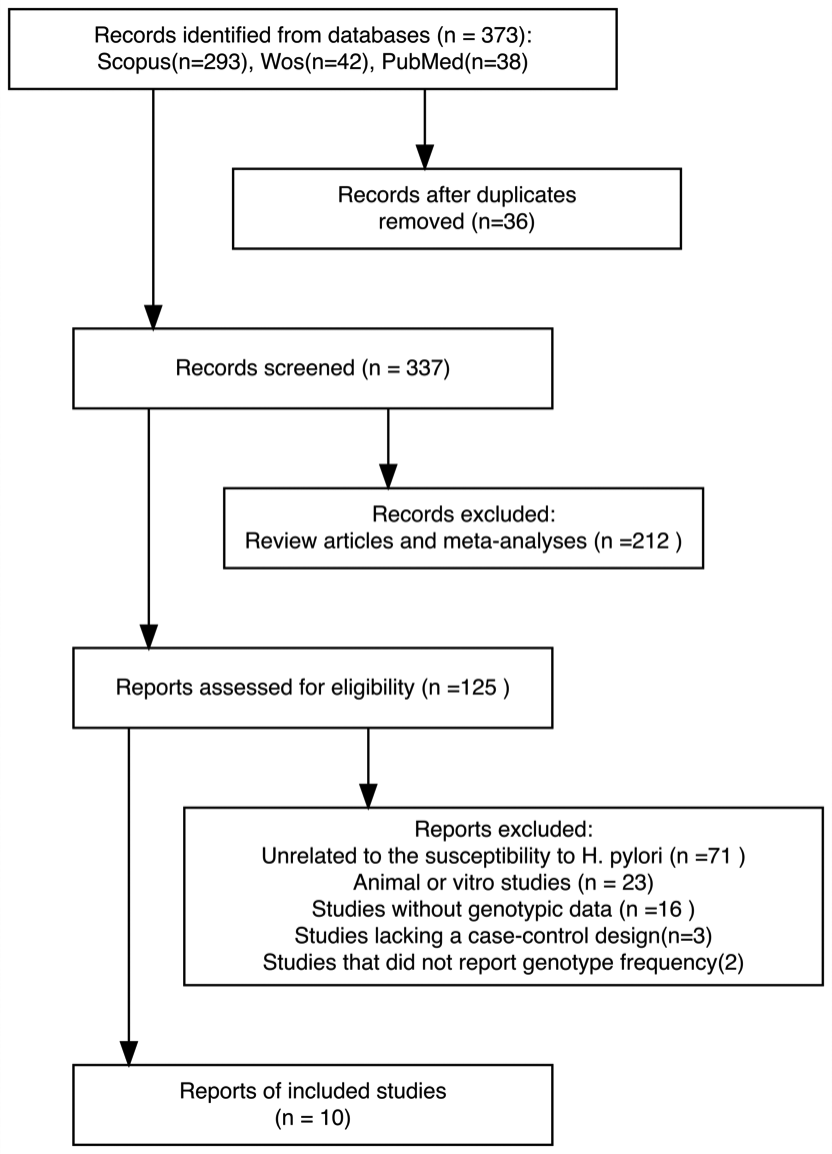
The flow diagram of study selection and identification for the meta-analysis.

A total of 373 records were initially identified through systematic searches of PubMed, Scopus, and Web of Science databases. After the removal of duplicates and the screening of titles and abstracts, 10 case–control studies met the predefined eligibility criteria and were included in the final analysis. The study selection process is illustrated in Figure 1.

Among the included studies, five investigated the TLR2 rs3804099 single nucleotide polymorphism (SNP), while the remaining five focused on the TLR2 del –196 to –174 promoter polymorphism. These studies provided detailed data on sample sizes, genotype distributions, demographic characteristics, and HWE status (Table 1). In total, data from 4,521 participants were analyzed, comprising 2,523 individuals infected with *H. pylori* and 1,998 uninfected controls.

**Table 1:** Attributes of all case–control investigations included in the meta-analysis.

Authors	Year	Continent (Country)	Age	Gender		Size of the sample	Genotype type frequency (Case)			Genotype type frequency (Control)		
				HP(+)	HP(-)							
			HP(+)/HP(-)	(M/F)	(M/F)	HP(+)/HP(-)	AA	AG	GG	AA	AG	GG
**TLR2 del** –**196 to** –**174**												
Lourenço^1^	2020	South America (Brazil)				619/233	481	127	11	159	65	9
Habibzadeh^4^	2017	Asia (Iran)				55/45	42	9	4	26	15	4
Tahara^5^	2010	Asia (Japan)				123/92	53	54	16	41	39	13
Hishida^6^	2010	Asia (Japan)				699/937	304	316	79	418	414	105
Rodrıguez^7^	2013	Asia (China)				190/94	81	90	19	46	39	6
**TLR2 rs3804099**												
Tongtawee^9^	2018	Asia (Thailand)	-	-	-	204/196	125	28	51	131	41	24
Mirkamandar^10^	2018	Asia (Iranian)	-	-	-	225/125	84	105	36	56	39	30
Tongtawee^11^	2018	Asia (Thailand)	46 ± 1.5/ 42 ± 2.5	71/13	65/131	204/196	126	27	51	131	41	24
Eed^12^	2020	Asia (Saudi Arabia)	45 ± 17.7/42 ± 22.3	117/93	41/39	210/80	171	24	15	65	8	7
Kalkanlı, Tas^13^	2020	Asia (Turkey)	47.7 ± 12 51.2 ± 12	83/122	84/111	205/195	28	99	78	18	75	102

### Meta-analysis findings

Pooled analyses under various genetic models – allelic, dominant, recessive, homozygous, and heterozygous – were conducted for both polymorphisms. For the TLR2 del –196 to –174 variant, no significant association with *H. pylori* infection risk was detected across any of the models examined. Specifically, the pooled ORs and 95% CIs were as follows: allelic model (A vs. T), OR = 1.04 (95% CI: 0.81–1.35); dominant model (AA vs. AT+TT), OR = 1.14 (95% CI: 0.81–1.59); recessive model (TT vs. AT+AA), OR = 0.96 (95% CI: 0.74–1.24); homozygote model (AA vs. TT), OR = 1.09 (95% CI: 0.73–1.64); and heterozygote model (AT vs. TT), OR = 1.02 (95% CI: 0.78–1.35).

**Figure 2:**
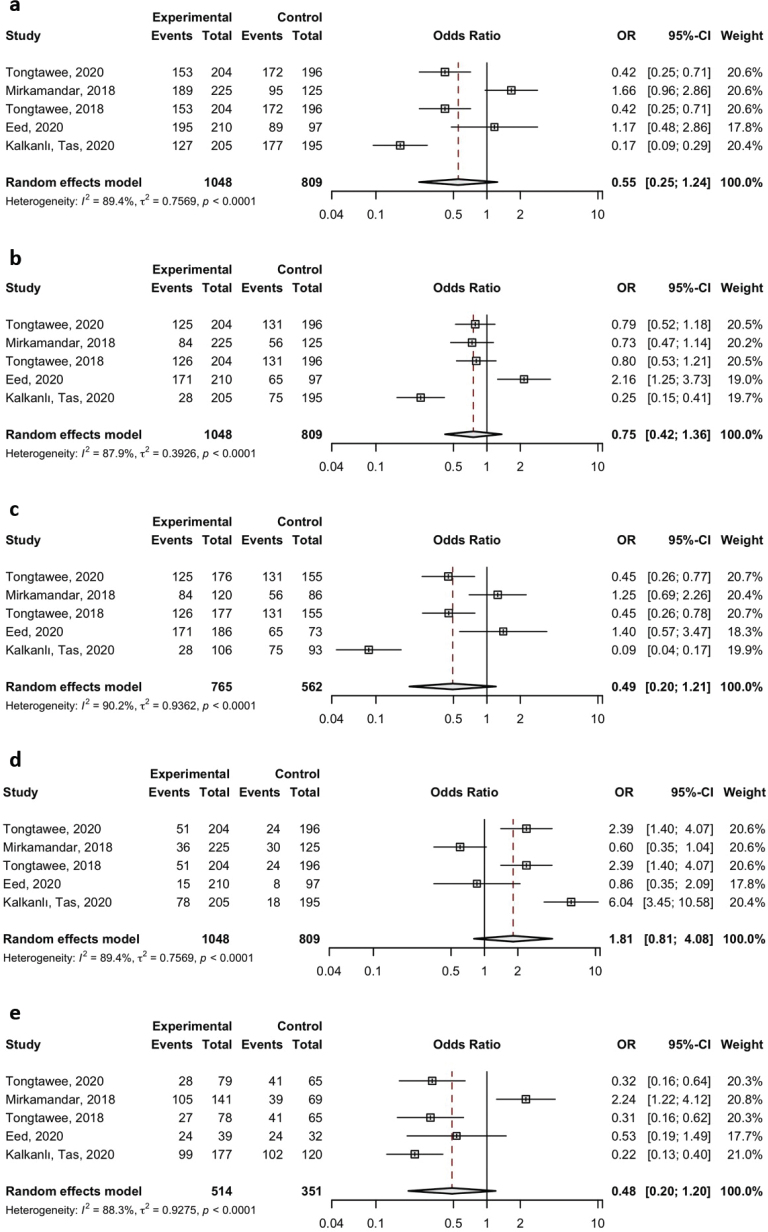
Meta-analysis of the association between TLR2 rs3804099 polymorphism and the risk of *H. pylori* infection was analyzed under the dominant (A vs. T) (a), recessive (AA vs. AT+TT) (b), allelic (AA vs. TT) (c), homozygote (TT vs. AT+AA) (d), and heterozygote (AT vs. TT) (e) genetic models.

**Figure 3:**
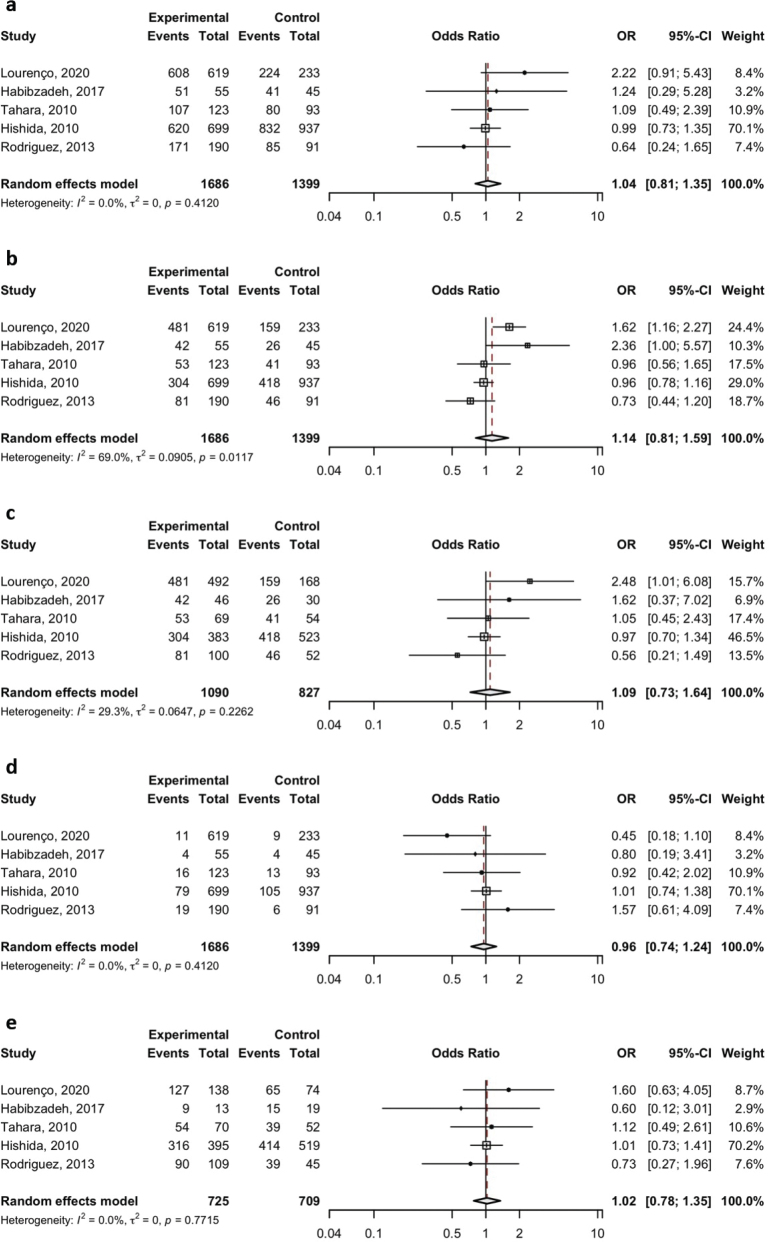
Meta-analysis of the association between TLR2 del –196 to –174 polymorphism and the risk of *H. pylori* infection was analyzed under the dominant (A vs. T) (a), recessive (AA vs. AT+TT) (b), allelic (AA vs. TT) (c), homozygote (TT vs. AT+AA) (d), and heterozygote (AT vs. TT) (e) genetic models.

Similarly, no statistically significant associations were observed for the TLR2 rs3804099 polymorphism under any of the genetic models: allelic model (A vs. T), OR = 0.55 (95% CI: 0.25–1.24); dominant model (AA vs. AT+TT), OR = 0.75 (95% CI: 0.42–1.36); recessive model (TT vs. AT+AA), OR = 1.81 (95% CI: 0.81–4.08); homozygote model (AA vs. TT), OR = 0.49 (95% CI: 0.20–1.21); and heterozygote model (AT vs. TT), OR = 0.48 (95% CI: 0.20–1.20). These results are summarized in Table 2 and graphically represented in Figures 2 and 3.

**Table 2:** Meta-analysis results of TLR2 gene polymorphisms and the risk of *H. pylori* infection.

Type of Toll-like receptor	Comparison	*N*	Association analysis			Heterogeneity analysis		Egger’s test of publication bias
			OR	95% CI	*P*	P	I²%	*P*
**TLR2 del** –**196 to** –**174**	A vs. T	5	1.04	(0.81, 1.35)	0.746	0.412	0.00%	0.653
	AA vs. AT+TT	5	1.14	(0.81, 1.59)	0.457	0.012	69.00%	0.586
	AA vs. TT	5	1.09	(0.73, 1.64)	0.671	0.226	29.30%	0.607
	TT vs. AT+AA	5	0.96	(0.74, 1.24)	0.746	0.412	0.00%	0.653
	AT vs. TT	5	1.02	(0.78, 1.35)	0.861	0.771	0.00%	0.815
**TLR2 rs3804099**	A vs. T	5	0.55	(0.25, 1.24)	0.151	< 0.001	89.44%	0.666
	AA vs. AT+TT	5	0.75	(0.42, 1.36)	0.347	< 0.001	88.94%	0.769
	AA vs. TT	5	0.49	(0.20, 1.21)	0.121	< 0.001	90.17%	0.870
	TT vs. AT+AA	5	1.81	(0.81, 4.08)	0.151	< 0.001	89.44%	0.666
	AT vs. TT	5	0.48	(0.20, 1.20)	0.116	< 0.001	88.30%	0.945

OR: odds ratio; CI: confidence interval.

### Heterogeneity and publication bias

The analysis of heterogeneity revealed substantial between-study variability for most genetic models involving the TLR2 rs3804099 polymorphism (*I*² > 88%, *P* < 0.001), whereas the TLR2 del –196 to –174 polymorphism exhibited low to moderate heterogeneity in most comparisons. Consequently, a random-effects model was employed where appropriate.

Publication bias was assessed using Egger’s test and visual inspection of funnel plots (Supplementary Files S2–S3). No significant asymmetry was detected (*P* > 0.05 across all models), indicating a low likelihood of publication bias (Table 2).

To further explore the robustness of the results and identify potential sources of heterogeneity, we performed leave-one-out sensitivity analyses for each genetic model and polymorphism (Supplementary File S4). While no single study disproportionately influenced the results for the del –196 to –174 variant, the rs3804099 analyses revealed that exclusion of Mirkamandar et al. (15) substantially reduced or eliminated heterogeneity and yielded statistically significant associations under certain models. This finding suggests that the study by Mirkamandar et al., which was the only one to provide cagA-stratified genotype data, had a uniquely strong influence on the pooled results for rs3804099, likely due to its specific population and bacterial strain characteristics.

## Discussion

This meta-analysis sought to elucidate the relationship between two extensively studied Toll-like receptor 2 (TLR2) polymorphisms – rs3804099 and del –196 to –174 – and susceptibility to *H. pylori* infection. Although biological plausibility and previous findings suggested a potential role for TLR2 in host immune responses to *H. pylori*, our pooled analyses across multiple genetic models did not reveal any statistically significant association between either polymorphism or infection risk.

The rs3804099 variant, although a synonymous SNP, has been proposed to influence immune regulation via potential effects on mRNA splicing, stability, or translation efficiency (15). The del −196 to −174 polymorphism, located in the gene’s promoter region, is known to affect transcriptional activity and, consequently, receptor expression levels. It was thus hypothesized that these variants could modulate host–pathogen interactions by altering TLR2 expression or function. Indeed, Mirkamandar et al. reported that the CT genotype at rs3804099 was significantly more prevalent in *H. pylori*-infected individuals, especially among those harboring cagA-positive strains, compared with healthy controls in an Iranian cohort (15). This observation suggested a genotype-dependent modulation of host immune responses, potentially predisposing certain individuals to more severe *H. pylori*-related outcomes such as peptic ulcer. However, the results of our meta-analysis, which includes data from diverse populations, do not support a consistent association. One possible explanation for this discrepancy could be population stratification and genetic heterogeneity among the included cohorts. For instance, while the Iranian population showed enrichment of the CT genotype in infected subjects, such findings were not replicated in East Asian or Turkish cohorts. This aligns with Moura et al., who observed no significant association between TLR2 polymorphisms and *H. pylori* infection or duodenal ulcer in Brazilian children, despite examining multiple TLR genes, including TLR2, TLR4, and TLR5. These findings underscore the complex and likely population-specific nature of TLR2 genetic contributions to *H. pylori* pathogenesis (29).

Another factor contributing to the variation across studies may be the interaction between host genotype and bacterial virulence determinants. The influence of TLR2 variants could be more evident in infections involving highly virulent *H. pylori* strains, particularly those expressing CagA or VacA. Mirkamandar et al. noted that the rs3804099 CT genotype was associated with a higher prevalence of CagA-positive infection, supporting the notion that specific host–pathogen combinations may exacerbate disease severity (15). Unfortunately, most studies included in our meta-analysis did not stratify data by *H. pylori* strain subtype, precluding a more granular evaluation of this interaction.

In terms of immune response, TLR2 activation by *H. pylori* components, such as lipoproteins and peptidoglycans, leads to NF-κB activation and subsequent cytokine production (30). Functional studies have shown that certain TLR2 genotypes can influence cytokine profiles, including TNF-α, IL-8, and IL-10, upon bacterial stimulation (31, 32). While this provides a mechanistic basis for genetic modulation of infection outcomes, the absence of a consistent epidemiological association in our analysis suggests that these effects may be context-dependent or modulated by additional genetic and environmental factors.

This meta-analysis provides a comprehensive synthesis of the current evidence regarding the association between *TLR2* polymorphisms and susceptibility to *H. pylori* infection. Several strengths should be noted, including strict adherence to PRISMA guidelines, broad literature coverage, and robust evaluation of heterogeneity and publication bias through multiple complementary techniques.

However, several limitations must be acknowledged to contextualize our findings and guide future research. First, both *TLR2* rs3804099 and del –196 to –174 polymorphisms were investigated in only five independent studies each, inherently limiting the statistical power to detect modest associations or to perform formal subgroup or meta-regression analyses. Although variables such as ethnicity and *H. pylori* virulence determinants (e.g. CagA status) were recognized a priori as potential effect modifiers, the included studies lacked sufficient ethnic and geographic diversity, as well as stratified reporting. Of the 10 total studies, only Mirkamandar et al. (15) provided genotype data stratified by CagA status, and only the study by Lourenço et al. (18) was conducted in a non-Asian population. The majority of included studies were conducted in Asian populations, thereby limiting the ability to explore regional variation or perform stratified analyses by ethnicity.

It is also important to note that nine of the 10 studies included in this meta-analysis originated from Asian populations, reflecting both the higher epidemiological burden of *H. pylori* infection and the predominance of molecular genetic research in these regions. This geographical imbalance inevitably limits the generalizability of our findings to non-Asian populations. Published population-genetic data, including those from the 1,000 Genomes and HapMap projects, indicate clear inter-ethnic variability in TLR2 allele frequencies – particularly for rs3804099 and the del −196 to −174 variant – with minor alleles occurring more frequently in East Asian than in European or African cohorts. Such differences may influence receptor expression levels or downstream signaling responsiveness, potentially modifying infection susceptibility. Therefore, while our results provide robust evidence for Asian populations, extrapolation to other regions should be made cautiously, and further studies involving Western and African populations are warranted to confirm the global relevance of these associations.

While no substantial heterogeneity was observed for the del –196 to –174 polymorphism across any genetic model, considerable between-study heterogeneity was detected in the rs3804099 analysis. To further assess the robustness of the findings and investigate potential sources of heterogeneity specific to rs3804099, we conducted leave-one-out sensitivity analyses across all genetic models related to this variant (see Supplementary File S4).

Notably, under the heterozygote model for rs3804099 (Genetic Model E), exclusion of Mirkamandar et al. (15) resulted in a statistically significant association (OR = 0.30, 95% CI: 0.21–0.42) and completely eliminated heterogeneity (*I*² = 0%, *P* = 0.529). A similar effect was observed under the dominant model (Model A), where removing the same study yielded a significant association (OR = 0.40, 95% CI: 0.21–0.79) and reduced heterogeneity from 88.3% to 79.5% (*P* = 0.002). These findings suggest that Mirkamandar et al. had a disproportionately large influence on the pooled estimates and heterogeneity under certain genetic models for rs3804099 – likely due to its unique stratification by CagA status. Although this observation cannot substitute for formal subgroup analysis, it provides indirect support for the hypothesis that CagA-positive *H. pylori* strains may modulate the association between rs3804099 and infection susceptibility. This aligns with the biological rationale that TLR2 plays a key role in mediating host–pathogen interactions, especially in response to virulent *H. pylori* strains. However, due to the lack of comparable stratified data in other studies, we were unable to explore this relationship more deeply through subgroup or meta-regression analysis.

Additionally, important potential confounding variables – such as dietary habits, smoking status, co-infections, socioeconomic status, and environmental exposures – were not consistently reported across studies, which further limited interpretability. Likewise, differences in *H. pylori* diagnostic methods (e.g. histology, PCR, rapid urease test, and serology) may have introduced misclassification bias, potentially affecting the accuracy of infection status classification and contributing to heterogeneity in effect estimates. Although subgroup analysis based on diagnostic method was not feasible due to insufficient or inconsistent reporting, we acknowledge this heterogeneity as a limitation of our study. This emphasizes how crucial it is for future genetic association research to employ standardized and verified diagnostic procedures.

Despite these limitations, our sensitivity analysis adds an important dimension to the interpretation of pooled results, helping to identify specific studies that disproportionately affect outcomes and potentially introduce heterogeneity. The fact that the only study reporting CagA-stratified genotypes was also the primary contributor to heterogeneity underscores the importance of future research incorporating bacterial virulence markers and host characteristics. Future investigations should present genotype data stratified by ethnicity and bacterial virulence factors, thereby enabling more refined analyses of gene–environment and gene–pathogen interactions.

In conclusion, although this meta-analysis did not reveal a consistent overall association between TLR2 rs3804099 or del −196 to −174 polymorphisms and *H. pylori* susceptibility, the results suggest that these variants may influence infection risk in specific genetic or bacterial contexts. Future investigations with larger, ethnically diverse cohorts and standardized reporting of bacterial virulence markers are warranted to further elucidate the nuanced role of TLR2 variability in host susceptibility and clinical outcomes.

## Conclusion

In this meta-analysis, we did not observe statistically significant associations between the TLR2 rs3804099 and del –196 to –174 polymorphisms and susceptibility to *H. pylori* infection across the included studies. However, these findings should be interpreted with caution due to key limitations, including the small number of available studies, high between-study heterogeneity (especially for rs3804099), and lack of stratified or subgroup analyses. Sensitivity analysis suggested a potential role for *CagA* status in modifying the association with rs3804099, but this finding remains hypothesis-generating rather than conclusive. Overall, the current evidence does not support a consistent genetic effect of these variants; nevertheless, further well-designed studies with larger and more diverse populations, bacterial genotyping, and stratified analyses are required to clarify the context-dependent nature of TLR2’s contribution to *H. pylori* infection risk.

## ORCID

Duygu Kirkik https://orcid.org/0000-0003-1417-6915

Sevgi Demircioğlu https://orcid.org/0000-0003-3900-0713

Sevgi Kalkanli Taş https://orcid.org/0000-0001-5288-6040
